# A prebiotic-based biostimulant enhances growth parameters, photosynthetic efficiency, and grain yield in rice (*Oryza sativa* ssp. *japonica*)

**DOI:** 10.3389/fpls.2026.1832147

**Published:** 2026-06-17

**Authors:** Karina Medina-Jiménez, Brett Hale, Carolina Cerquera-Hernández, Reinier Gesto-Borroto, Argelia Lorence

**Affiliations:** 1 Arkansas Biosciences Institute, Arkansas State University, Jonesboro, AR, United States; 2 AgriGro, Inc, Doniphan, MO, United States; 3 Department of Chemistry and Physics, Arkansas State University, Jonesboro, AR, United States

**Keywords:** biostimulant, FoliarBlend, phenotyping, photosynthesis, prebiotic, rice

## Abstract

Biostimulants constitute an emerging class of biological inputs with potential to boost crop yield in a sustainable manner. However, such products must be tested rigorously to unravel complex and oftentimes discrete modes of action, which is crucial for optimizing product placement and maximizing grower return on investment. This study combined high-throughput phenotyping of seedling establishment, assessment of vegetative and reproductive development, and quantification of photosynthetic efficiency to determine the impact of FoliarBlend, a prebiotic-based biostimulant, on the rice var. Kitaake (Oryza sativa ssp. japonica) from seed germination through seed production. Automated, non-invasive phenotyping revealed that FoliarBlend-treated seedlings displayed consistently elevated projected leaf area, convex hull area, caliper length, and compactness from 5 to 14 days after germination (DAG). The treatment did not introduce additional physiological stress, as evidenced by levels of chlorophyll fluorescence, relative water content, and estimation of chlorotic leaf surface area. Manual phenotyping from 21 to 82 DAG revealed that FoliarBlend application had paradoxical effects on vegetative and reproductive development, decreasing leaf and tiller number while increasing plant height, panicle number, above and belowground biomass, and ultimately grain yield. Furthermore, assessment of photosynthetic parameters highlighted improved Photosystem II (PSII) efficiency and chlorophyll content in treated plants from 49 to 82 DAG, along with an elevation in the quantum yield of non-regulated energy dissipation in PSII. These outcomes indicate that FoliarBlend positively impacts rice seedling establishment, reproductive growth, photosynthetic efficiency, and grain yield under controlled conditions, posing significant implications for enhancing rice production.

## Introduction

Conventional agriculture has been vital in supporting the global population, marking significant advances in food and fiber production efficiency (Gollin et al., 2021). However, the intensification of agricultural practices has also brought to light the potential for adverse environmental impacts. These include degradation of soil quality, water scarcity, and loss of biodiversity, which become exacerbated when cultivation methods are not employed responsibly or become excessively reliant on traditional chemical inputs (Horrigan et al., 2002; van Der Werf et al., 2020). In response, there has been a pronounced shift in consumer and regulatory demand towards more sustainable agricultural practices (Piñeiro et al., 2020; Kassam and Kassam, 2021; Köninger et al., 2021), sparking interest in alternative inputs such as biofertilizers, biopesticides, and biostimulants (Du Jardin, 2015). Among these, biostimulants have experienced particularly notable growth in recent years. The Biostimulant Industry Workgroup defines a biostimulant as “a substance(s), microorganism(s), or mixtures thereof, that, when applied to seeds, plants, the rhizosphere, soil, or other growth media, act to support a plant’s natural nutrition processes independently of the biostimulant’s nutrient content, thereby improving nutrient availability, uptake, or use efficiency, tolerance to abiotic stress, and consequent growth, development, crop quality, or yield” (Biological Products Industry Alliance and The Fertilizer Institute, 2022). Furthermore, the biostimulant sector’s market size is projected to increase from USD 3.9 billion in 2023 to USD 6.8 billion by 2028 at a compound annual growth rate of 11.8% (Markets and Markets, 2023). Despite this rapid expansion, the biostimulant industry faces challenges, including the lack of a unified definition and regulatory framework, which are critical for integration into mainstream agricultural practices (Biological Products Industry Alliance and The Fertilizer Institute, 2022). Such guidelines must address product claims/mode of action (e.g., growth enhancement and increased survival), which may be subtle or even inconsistent across production environments and plant genotypes (Hellequin et al., 2020; Carletti et al., 2021).

High-throughput phenotyping (i.e., phenomics) has been used widely in plant breeding programs to link organism genotype to phenotype (Campbell et al., 2018) and offers potential to detect and quantify discrete parameters of biostimulant performance (Petrozza et al., 2014; Rouphael et al., 2018). This approach employs advanced automation technologies to screen large numbers of plants quickly and efficiently, facilitating the collection of vast phenotypic data while minimizing labor costs and reducing the potential for human error (Fahlgren et al., 2015; Campbell et al., 2018). Moreover, high-throughput phenotyping is inherently non-destructive, allowing for the continuous monitoring of plant growth and development over time. Commonly assessed metrics include plant height, area, and architecture, leaf color, chlorophyll content, photosynthetic efficiency, relative water content, and resilience to biotic and abiotic stressors (Campbell et al., 2018), all of which are critical for assessing the influence of biostimulants for economic yield preservation (Rouphael et al., 2018 and references therein). Thus, with the growing adoption of biostimulants in conventional agriculture, high-throughput phenotyping (and other phenotyping methodologies) will be instrumental in elucidating complex genotype-phenotype-environment interactions that translate into sustainable, yet profitable, agriculture productivity (Pieruschka and Schurr, 2019).

Rice (*Oryza sativa* L.) is a staple food for nearly half of the global population, contributing to approximately 19% of worldwide caloric intake (Elert, 2014; Prasad et al., 2017). With an expected annual increase in rice consumption of 1% (Prasad et al., 2017), rice cultivation faces both inherent challenges and environmental concerns. These challenges include abiotic stressors such as heat, drought, and soil salinity (Dar et al., 2021), biotic stress from pests and diseases (Ansari et al., 2015; Singh et al., 2020), and environmental pollution issues related to fertilizer runoff and greenhouse gas emissions (Prasad et al., 2017 and references therein). Coupled with the effects of climate instability, these factors pose significant hurdles to increasing rice production to meet the demands of a growing population (Ray et al., 2013; Bailey-Serres et al., 2019). Biostimulants have emerged as valuable tools in addressing such issues. For example, seaweed-based biostimulants (predominantly derived from *Kappaphycus*, *Eucheuma*, and *Ascophyllum* spp.) have been evidenced to improve rice growth metrics upon exposure to drought (Abdel Megeed et al., 2021), fungicides (Banakar et al., 2022), *Magnaporthe oryzae* (causal agent of rice blast) (Sahana et al., 2022), and transplantation (Arun et al., 2019), as well as under normal growing conditions (Sharma et al., 2017; Layek et al., 2018). Additionally, other biostimulant formulations have shown promise in reducing dependence on conventional fertilizers (Johnson and Puthur, 2021; Mulyatni et al., 2018; Hem and Pang, 2017; Wang et al., 2022; Hao et al., 2024), mitigating salinity (Khan et al., 2018; Pérez-Domínguez et al., 2022), and alleviating heat stress (Quintero-Calderon et al., 2021), thereby enhancing biomass accumulation and grain yield. The identification and utilization of consistent and effective biostimulants holds considerable potential for surmounting the challenges associated with rice production.

**Figure 1:**
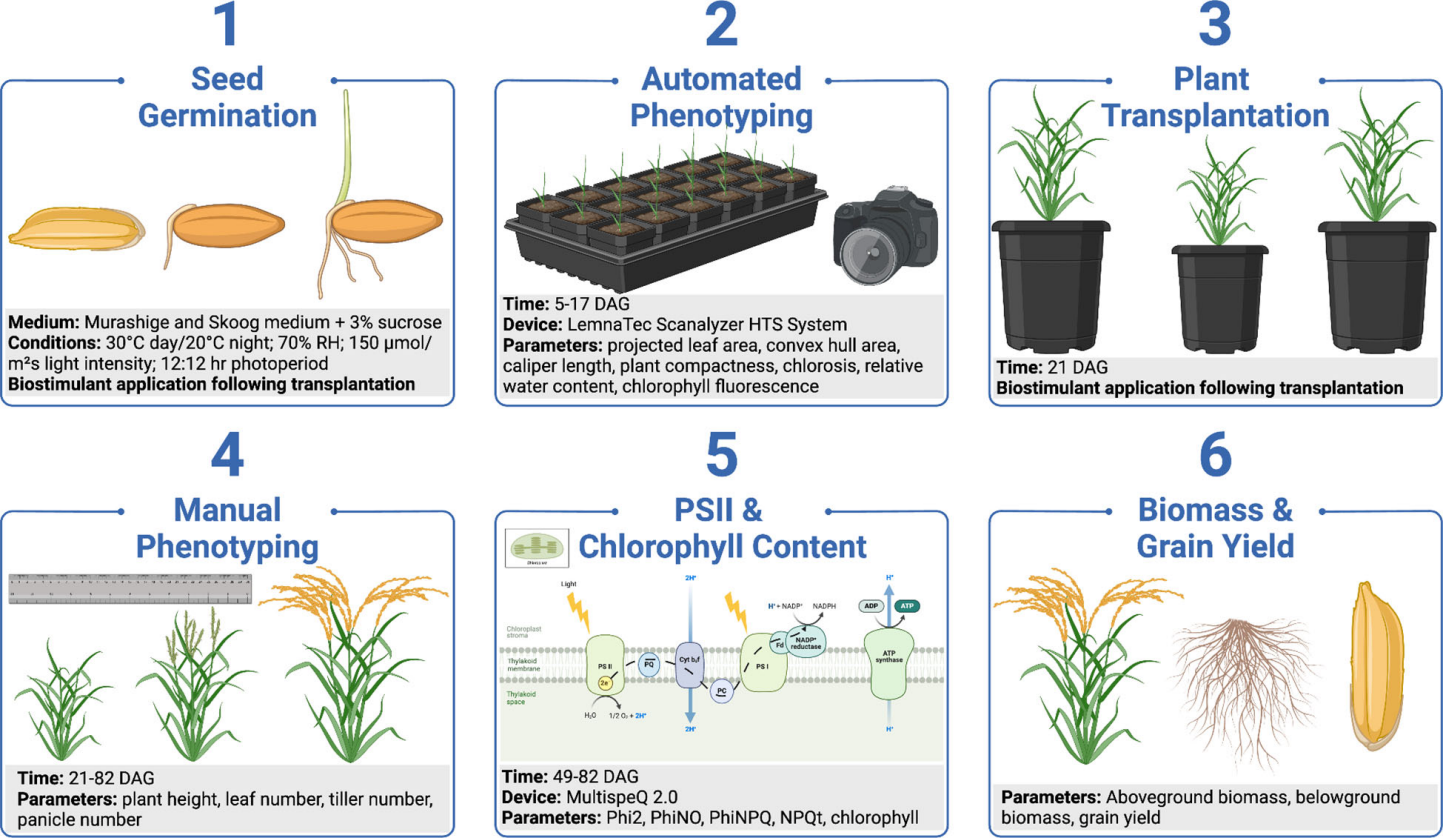
Schematic illustration of the study design.

FoliarBlend (AgriGro, Inc; Doniphan, MO, USA) is a prebiotic-based biostimulant deployed globally in rice production systems (https://agrigro.com/blogs/research). However, apart from grain yield, detailed phenotype characterization of the plant’s response to the product administration has not been reported. To this end, the objective of the present study was to evaluate the effects of FoliarBlend on rice growth and physiology in a controlled environment to gain mechanistic insights regarding this biostimulant, and inform more precise application strategies in future studies. The research was conducted by the Arkansas State University Phenomics Core Facility (https://www.astate.edu/a/abi/core-facilities/phenomics-core/phenomics-core-facility), a cost-recovery core facility housed at the Arkansas Biosciences Institute. Rice seeds were first germinated in a semi-solid medium and subsequently transplanted to soil, at which time the prebiotic was applied to the designated treatment group (**Figure 1**). Several parameters of seedling establishment and physiological stress level were detected and quantified with a high throughput phenotyping system. The plants were then transferred to larger pots, with the treated group receiving a second application of FoliarBlend. Measures of vegetative and reproductive growth were next determined via manual phenotyping, and photosynthetic efficiency and chlorophyll content measured with a handheld spectrophotometer. Above- and below-ground biomass, as well as grain yield, were calculated at the conclusion of the study. This work demonstrates the effectiveness of FoliarBlend to enhance rice growth under controlled conditions and further accentuates the value of phenotyping in understanding biostimulants modes of action.

## Materials and methods

### Plant material and growth conditions

This study utilized the rice var. Kitaake (*Oryza sativa* ssp. *japonica*). Seeds (*n* = 135) were surface-sterilized twice using 70% ethanol, 50% bleach (7.4% NaClO), and 0.05% Tween 20 (Fisher Scientific, Hampton, NH, USA). Each sterilization step lasted for 1 minute, and the seeds were rinsed subsequently with sterile water to ensure complete removal of sterilizing agents (six washes with 50 mL of sterile water each). Next, seeds were placed onto sterile Phytatrays (Cat #P1552; Sigma-Aldrich; Burlington, MA, USA) containing Murashige and Skoog semi-solid medium (Murashige and Skoog, 1962) supplemented with 3% sucrose (Cas #57-50-1; Fisher Scientific). Seed incubation was carried out in an environment-controlled Conviron chamber (Winnipeg, Canada) set to a diurnal temperature regime of 30°C during the day and 20°C at night. The chamber maintained 70% relative humidity and a light intensity of 150 µmol/m²s at a 12:12-h photoperiod.

After one week, 30 seedlings were selected based on growth and vigor for high-throughput plant phenotyping experiments and transplanted into 85x75 mm Quick Pot 15 RW containers (Herkuplast; Kubern, Germany). Growth conditions after transplanting were as cited in the previous paragraph. The growing medium encompassed a 1:1 mixture of Sun Gro Seed Germination Mix and topsoil. To this soil mixture, 30 g of the following supplements were uniformly added per gallon: High Yield dusting sulfur (Voluntary Purchasing Group; Bonham, TX, USA), Scotts Micro-max fertilizer (Scotts; Maryville, OH, USA), and Ironite (Ironite Product Company; Scottsdale, AZ, USA). The same amount of growing medium was added to each container. Trays were weighed on the day of transplantation, and both the treatment and control groups were watered to 100% saturation with well water (full soil saturation took 1.2 L for an entire tray). At this time, the watering regimen for the treatment group also included the prebiotic FoliarBlend (AgriGro, Inc; Doniphan, MO, USA) at a concentration of 1 ml/kg of soil. Subsequent watering was performed every other day.

After 2 weeks, rice plants were transplanted into half-gallon pots. The fifteen plants in the treatment group received a second application of FoliarBlend at the defined concentration of 1 ml/kg of soil applied at the base of the plant per manufacturer guidelines. Control plants were watered solely with well water. To ensure uniform experimental conditions, all plants were watered with an equal volume to achieve 100% soil saturation.

### Image acquisition

Images of rice plants were acquired every two days from seedling establishment to 17 days after germination (DAG) using the LemnaTec Scanalyzer HTS System paired with LemnaControl software. This non-destructive, high-throughput phenotyping platform comprises a robotic arm equipped with four distinct high-resolution cameras. These cameras are specialized for capturing top-view images across multiple spectra: visible (RGB), fluorescence (FLUO), near-infrared (NIR) and far infrared (Acosta-Gamboa et al., 2016). In these experiments only the RGB, FLUO and NIR camera were used. The RGB imaging was performed using a Basler piA2400–17 gc CCD camera (Ahrensburg, Germany) with an image resolution of 2, 454 x 2, 056 pixels. For FLUO imaging, a Basler scA1600–14 gc CCD camera was employed with a 1, 624 x 1, 234 pixel resolution. The NIR imaging utilized a Goldeye GIGE P-008 SWIR camera from Allied Vision Technologies (Stadtroda, Germany), featuring a resolution of 320x256 pixels and a spectral sensitivity range between 900 and 1, 700 nm. To account for circadian rhythm, phenotyping was carried out at the same time of day (3.5 h after onset of daylight ± 30 min). Camera settings were configured automatically via the LemnaControl software.

### Image analysis

Images of rice plants (15 biological replicates x 2 treatments x 5-time points x 3 cameras; *n* = 450) were analyzed using LemnaGrid software largely following the pipeline described by Acosta-Gamboa et al. (2016). The analysis conducted with the RGB camera images involved the identification of all distinguishable leaves, followed by the calculation of the projected leaf area (cm², leaf area observed from above), the convex hull area (cm², area of the smallest convex polygon that encloses the plant), caliper length (cm; informative of plant height), and plant compactness (i.e., “bushiness”). Additionally, RGB images were color-classified with LemnaGrid, and the relative leaf area showing green versus detectable yellow coloration (chlorophyll loss or chlorosis) was determined.

In the analysis of NIR images, grayscale levels ranging from 0 (black) to 255 (white) were categorized into three bins, with the centers of these bins equally distributed across the entire grayscale range. This classification enabled the assessment of varying water content and distribution within the plant, with darker tones indicating higher water content. The FLUO camera images were processed using a similar binning approach on the red scale, divided into four categories to evaluate chlorophyll fluorescence levels. This division enabled the quantification of the relative leaf areas exhibiting zero, low, medium, and high levels of fluorescence.

All raw and processed images, along with their associated data, were stored in the LemnaDB PostgreSQL database. The quantitative data derived from these images was exported subsequently as CSV files and was analyzed in RStudio (v4.2.2) (RStudio Team, 2020).

### Plant growth measurements

Throughout the study, a comprehensive set of metrics was recorded manually to capture both vegetative and reproductive development of the rice plants, starting from 21 DAG until maturation at 82 DAG. These metrics included the number of leaves, number of tillers from the stem elongation stage to maturation, number of panicles from the milking stage to maturation, and plant height, measured from the soil line to the tip of the longest leaf. In addition, both above-ground and below-ground biomass (g) and seed yield (g) were quantified at the end of the study.

To assess biomass, physiologically mature plants were carefully uprooted from the substrate and dried in a greenhouse at 65°C for 2 weeks. The dry biomass of each plant was then measured using a digital scale. Following biomass determination, seeds were harvested from each plant and weighed separately to calculate seed yield.

### Plant photosynthetic measurements

Non-destructive assessments of photosynthetic efficiency were conducted weekly from 49 DAG at panicle initiation to 82 DAG at maturation, using a MultispeQ 2.0 instrument (Kuhlgert et al., 2016). This analysis included measurements of Phi2 (ΦII - effective quantum yield of Photosystem II [PSII]), PhiNO (ΦNO - quantum yield of non-regulated energy dissipation in PSII), PhiNPQ (ΦNPQ - quantum yield of regulated energy dissipation in PSII), NPQt (overall non-photochemical quenching), and relative chlorophyll content (Soil Plant Analysis Development [SPAD]). These measurements were taken in green, photosynthetically active leaf tissue up to the point of physiological maturity prior to senesence. The MultispeQ was calibrated prior to use according to manufacturer’s guidelines. Initial data processing was performed in the PhotosynQ web portal (www.photosynq.org) and was followed by analyses in RStudio (v4.2.2).

### Statistical analysis

Data analysis and visualization were performed in RStudio (v4.2.2). Prior to hypothesis testing, assumptions of normality and homogeneity of variance were assessed using Shapiro-Wilk (Shapiro and Wilk, 1965) and Levene’s (Levene, 1960) tests, respectively. The Shapiro-Wilk test was performed using the “shapiro.test” function in the R package *stats* (v4.2.2) and the Levene Test leveraging “levenetest’’ in the *car* package (v3.0-12) (Fox et al., 2012). In cases where assumptions were met (*p* > 0.05), Welch’s *t*-test (Welch, 1947) was employed to determine the statistical significance of treatment effects by implementing the function “t.test” in *stats*. Conversely, non-Gaussian/heteroscedastic data was evaluated with Wilcoxon rank sum tests (Mann and Whitney, 1947) using “wilcox.test” in *stats*. All analyses were performed independently per time interval, when applicable. Data visualization was performed with *ggplot2* (v3.4.2) (Wickham, 2016).

## Results

### Prebiotic application improved seedling establishment

**Figure 2:**
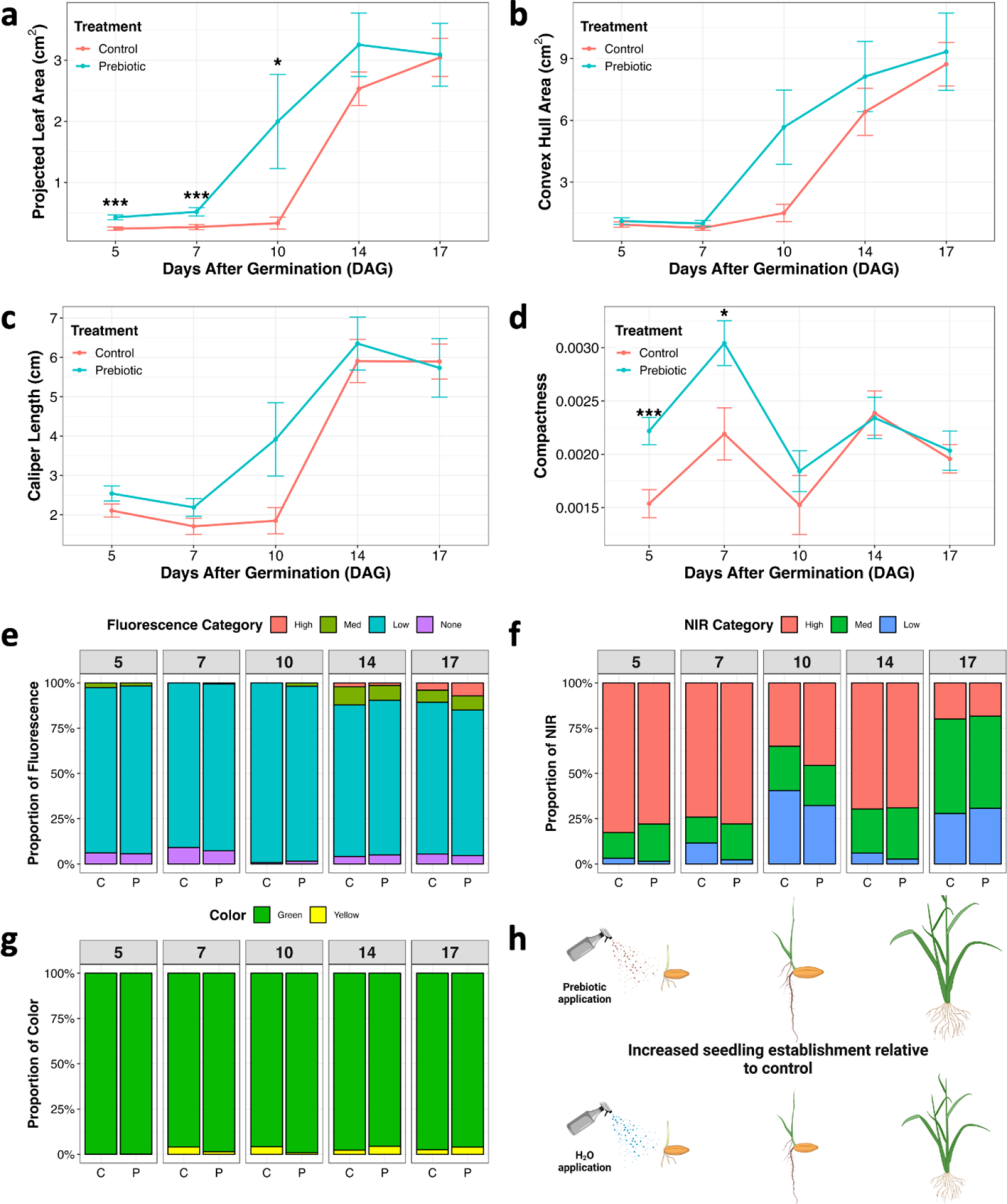
Assessment of seedling growth and physiological parameters with high-throughput phenotyping from 5-17 DAG. **(a)** Projected leaf area (cm²). **(b)** Convex hull area (cm²). **(c)** Caliper length (cm). **(d)** Compactness. **(e)** Proportion of relative chlorophyll fluorescence across treatments and time intervals, with values binned as high, medium, low, and none. **(f)** Proportion of near-infrared fluorescence (relative water content). Values represent high, medium, and low fluorescence. **(g)** Relative leaf area showing green versus yellow coloration. **(h)** Figure summary created with BioRender.com. Values are means ± standard error *p ≤ 0.05, **p ≤ 0.01, ***p ≤ 0.001.

The objective of this study was to evaluate impacts of the prebiotic FoliarBlend on rice physiology and development. Rice seeds were initially germinated in a semi-solid medium, transferred to soil, and treated with 1 ml/kg of FoliarBlend. Seedling growth parameters were assessed at 5, 7, 10, 14, and 17 days after germination (DAG) with visible (RGB) camera images obtained on the high-throughput phenotyping platform. Notably, treated seedlings exhibited a significant increase in projected leaf area by 5 DAG (W = 36, *p* = 9.97e-04 where W refers to the Wilcoxon rank-sum test), a trend that persisted at 7 (W = 33, *p* = 5.78e-04) and 10 DAG (W = 13, *p* = 0.03) and remained elevated compared to the control group at later intervals (**Figure 2a**). Convex hull area and caliper length did not change in the treatment group across all intervals (**Figures 2b, c**), although a slight decrease in caliper length was observed in the treatment group at 17 DAG (Control mean: 5.89 ± 0.44; Prebiotic mean: 5.73 ± 0.74). Compactness, defined as the ratio of projected leaf area to convex hull area (Acosta-Gamboa et al., 2016), displayed a pattern similar to projected leaf area, being significantly increased in the treatment at 5 (t = -3.71, *p*-value = 8.99e-04) and 7 (t = -2.63; *p*-value = 0.01) DAG and higher at two later intervals (**Figure 2d**). These findings collectively indicate that the prebiotic application accelerated growth in rice seedlings.

Metrics inferring plant stress were determined from fluorescence (FLUO), near-infrared (NIR), and RGB camera images from the high-throughput phenotyping platform. These measurements aimed to ascertain if the observed growth differences were a result of the treatment or potentially due to stress from transplantation or watering, and, at later intervals, to detect any changes in physiology attributable to the treatment. The FLUO images were used to estimate leaf-relative chlorophyll fluorescence, which is employed commonly as a proxy for abiotic stress response (Baker, 2008). In doing so, fluorescence proportions were binned as “high”, “medium”, “low”, or “none” on a per-plant basis. No statistical significance was observed across fluorescence categories or DAG, though elevated (High and Medium) chlorophyll fluorescence became more prevalent at later intervals (**Figure 2e**). The NIR imaging enabled the quantification of *in planta* water content and distribution (Acosta-Gamboa et al., 2016), with the binned categories (“high”, “medium”, and “low” water content) showing qualitative variation by DAG (**Figure 2f**). No significant differences were observed between the treatments at any interval. Furthermore, color classification was performed with the VIS images used for growth estimation, and the relative leaf area with green versus yellow coloring (indicative of chlorosis) was calculated. These proportions were similar between treatments at every interval except 14 DAG, where the treatment group demonstrated marginally elevated chlorosis (Green W = 95, Yellow W = 25, *p* = 0.02) (**Figure 2g**). Overall, prebiotic application improved seedling establishment independent of water content and detectable stress response (**Figure 2h**).

### Vegetative and reproductive development responded inversely to prebiotic application

**Figure 3:**
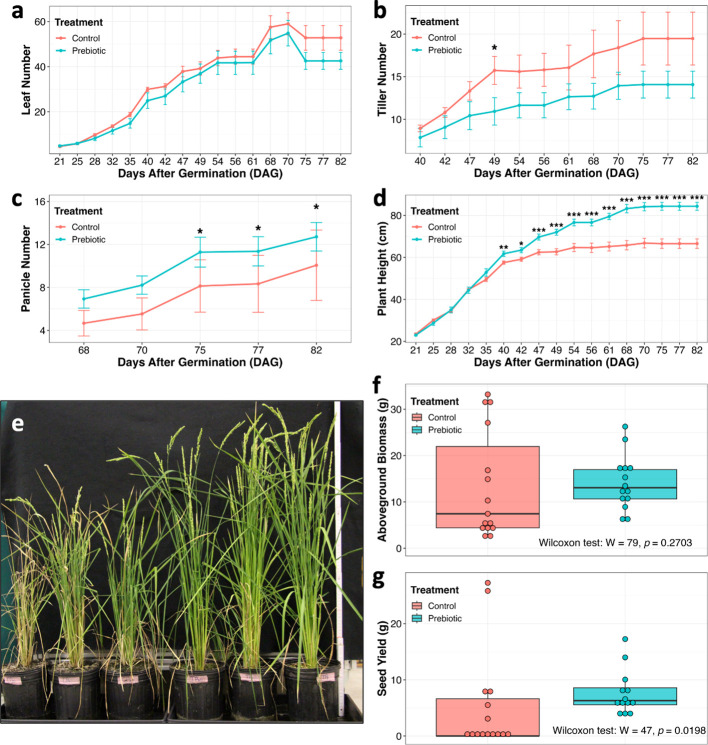
Manual phenotyping of vegetative and reproductive growth, aboveground biomass, and seed (grain) yield. **(a)** Leaf number from 21-82 DAG (transplantation to maturation). **(b)** Tiller number from 40-82 DAG (stem elongation stage to maturation). **(c)** Panicle number from 68-82 DAG (milk stage to maturation). **(d)** Plant height (cm) from 21-82 DAG. **(e)** Image of rice plants at 56 DAG. The three plants on the left represent the control group and the three on the right represent the treatment group. **(f)** Aboveground biomass (g dry weight) determined at the conclusion of the study. **(g)** Seed (grain) yield **(g)**. Values are means ± standard error, *p ≤ 0.05, **p ≤ 0.01, ***p ≤ 0.001.

At 21 DAG, plants were transplanted to larger pots and manually phenotyped until 82 DAG (maturation). This entailed periodic measurements of leaf number (21-82 DAG), tiller number (40-82 DAG; stem elongation-maturation), panicle number (68-82 DAG; milking stage-maturation), and plant height (21-82 DAG). During this period, control plants exhibited a slightly higher leaf number than those treated with FoliarBlend, though the difference was not statistically significant (**Figure 3a**). Similarly, tiller number was consistently lower in the treated group, with a significant reduction at 49 DAG (panicle initiation; W = 161, *p* = 0.01) and near-significant differences at 54 (W = 146, *p* = 0.08) and 56 (booting stage; W = 149.5, *p* = 0.05) DAG (**Figure 3b**). In contrast, panicle number was increased in treated plants at every interval, with statistically significant differences observed at 75 (W = 55.5, *p* = 0.03), 77 (W = 55.5, *p* = 0.03), and 82 (W = 59, *p* = 0.045) DAG (**Figure 3c**). Plant height was also notably affected by FoliarBlend, showing a significant increase from 40 DAG to the study’s end (**Figures 3d, e**). These results suggest that vegetative and reproductive growth responded differently to the prebiotic application.

Total aboveground biomass (dry weight) and seed yield were determined at the end of the study. FoliarBlend application led to a marginal, non-significant increase in biomass (Control mean: 13.5 ± 3.01; Prebiotic mean: 14.1 ± 1.57; W = 79, *p* = 0.27) (**Figure 3f**). However, a more pronounced effect was observed in seed yield. Notably, only 46.7% (7/15) of the control group produced seeds, compared to 100% (13/13) in the treatment group (**Figure 3g**). This resulted in a significant increase in seed yield for the treated plants (Control mean: 5.22 ± 2.36; Prebiotic mean: 7.7 ± 1.11; W = 47, *p* = 0.02) (**Figure 3g**). Therefore, the increase in reproductive tissues and plant height in the treatment group rendered heightened total aboveground biomass and seed yield.

### Treated plants displayed enhanced photosynthetic efficiency

**Figure 4:**
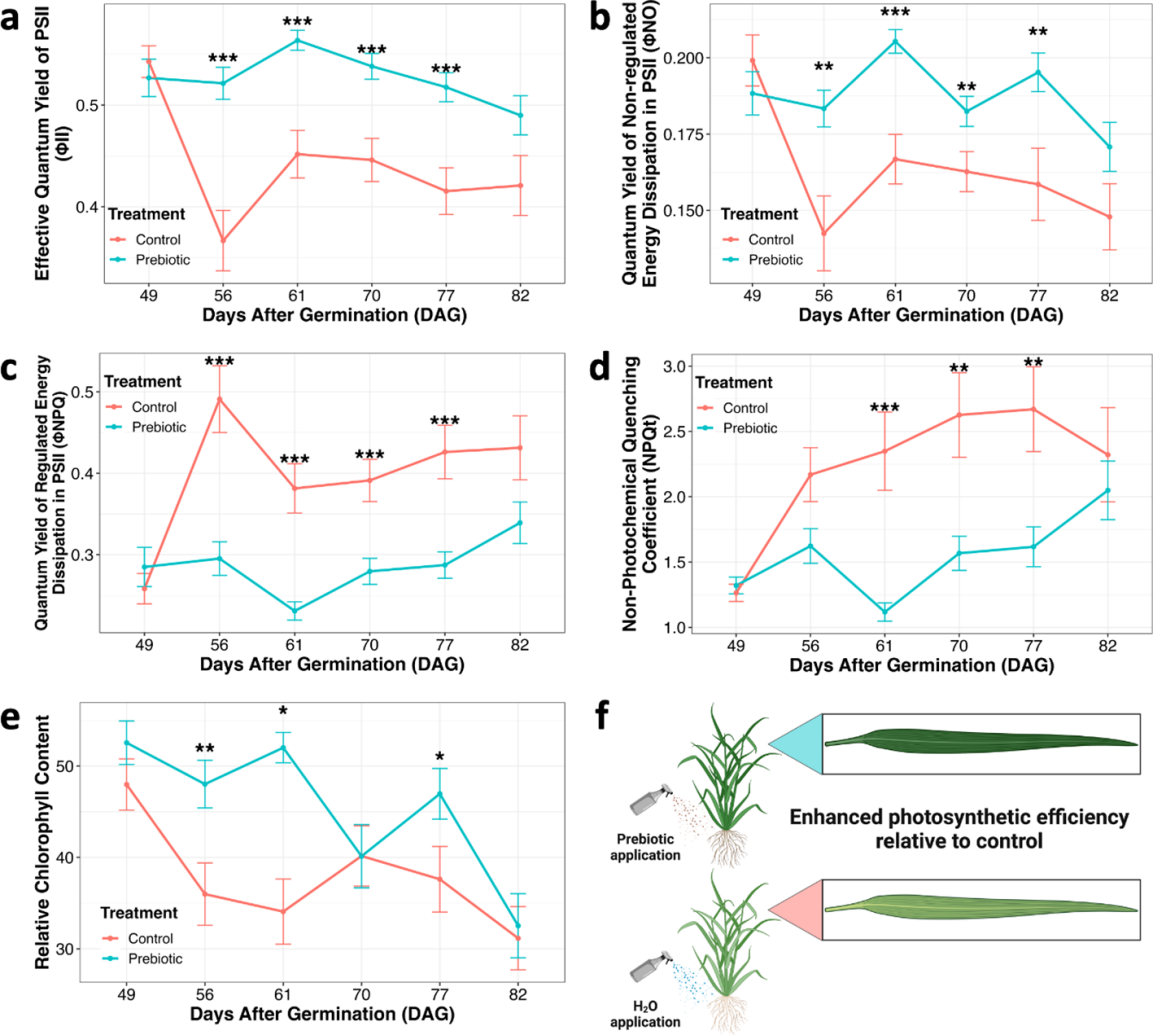
Estimation of photosynthetic efficiency and relative chlorophyll content from 49-82 DAG. (panicle initiation to plant maturation) **(a)** Phi2 (ΦII - effective quantum yield of Photosystem II [PSII]). **(b)** PhiNO (ΦNO - quantum yield of non-regulated energy dissipation in PSII). **(c)** PhiNPQ (ΦNPQ - quantum yield of regulated energy dissipation in PSII). **(d)** NPQt (non-photochemical quenching). **(e)** Relative chlorophyll content (SPAD). **(f)** Figure summary created with BioRender.com. Values are means ± standard error, *p ≤ 0.05, **p ≤ 0.01, ***p ≤ 0.001.

Complementary to above-ground developmental phenotyping, photosynthetic efficiency was assessed with a MultispeQ 2.0 spectrophotometer at 49 (panicle initiation), 56 (booting stage), 61 (flowering stage), 70 (dough phase), 77, and 82 DAG (maturation stages). Evaluated metrics included Phi2 (effective quantum yield of Photosystem II [PSII]), PhiNO (quantum yield of non-regulated energy dissipation in PSII), PhiNPQ (quantum yield of regulated energy dissipation in PSII), NPQt (non-photochemical quenching), and relative chlorophyll content. The treatment group exhibited increased Phi2 at five of the six intervals, with statistical significance at intervals 56 (W = 427, *p* = 2.92e-05), 61 (W = 473, *p* = 2.25e-05), 70 (W = 551.5, *p* = 8.43e-04), and 77 (W = 551.5, *p* = 8.43e-04) DAG (**Figure 4a**).

Surprisingly, a near-identical trend was observed for PhiNO, showing an increase at several intervals with statistical significance (56 W = 598, *p* = 0.007; 61 W = 493, *p* = 4.602e-05; 70 W = 627.5, *p* = 0.007; 77 W = 416, *p* = 0.004) (**Figure 4b**). These results suggest improved photochemical activity in treated plants as well as elevated passive light dissipation.

Furthermore, prebiotic application consistently reduced PhiNPQ and NPQt, which concomitantly inferred a decrease in regulated non-photochemical energy dissipation and overall enhanced photosynthetic efficiency. The former was reduced at five of six intervals with significance mirroring that of Phi2 and PhiNO (56 W = 1340, *p* = 1.24e-04; 61 W = 1593, *p* = 7.638e-07; 70 W = 1356.5, *p* = 4.81e-04; 77 W = 1003, *p* = 5.00e-04) (**Figure 4c**). NPQt was also reduced in treated plants at all but one interval, with significant reductions at 61 (W = 1312, *p* = 8.569e-07), 70 (W = 1088, *p* = 0.003), and 77 (W = 854, *p* = 0.003) DAG and a nearly significant reduction at 56 DAG (W = 792, *p* = 0.05) (**Figure 4d**). Relative chlorophyll content paralleled photosynthetic efficiency, being increased at nearly every interval upon FoliarBlend application, with significance observed at 56 (W = 599, *p* = 0.008), 61 (W = 692, *p* = 0.01), and 77 (W = 494, *p* = 0.04) DAG (**Figure 4e**). In summary, these findings collectively indicate that prebiotic application enhanced photosynthetic efficiency while also affecting the balance between regulated and non-regulated energy dissipation.

### Qualitative differences in root system architecture were inferred upon prebiotic application

**Figure 5:**
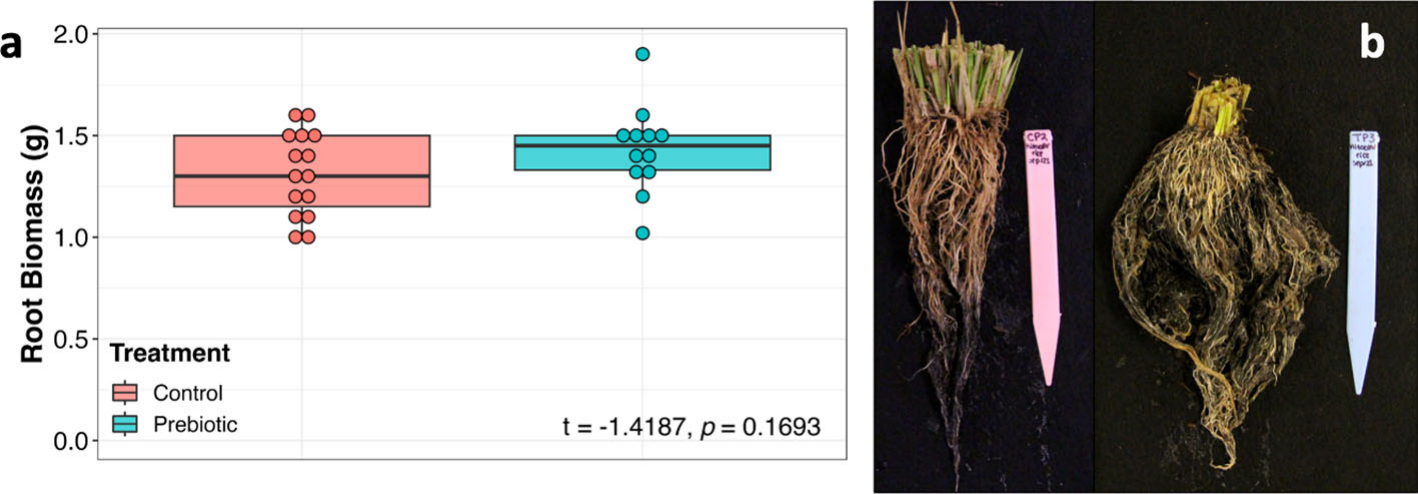
Comparison of belowground biomass and root system architecture between treatments **(a)** Root biomass (g dry weight). **(b)** Image of representative root morphologies for each treatment. The prebiotic-treated plants were demarcated by expansive lateral root formation relative to the control.

Consistent with aboveground biomass, root dry weight was measured and compared across treatments at the end of the study. A statistically insignificant increase was observed in treated plants (Control mean: 1.31 ± 0.05; Prebiotic mean: 1.43 ± 0.06; t = -1.42, *p* = 0.17) (**Figure 5a**). More notably, the root system architecture exhibited marked qualitative changes under prebiotic treatment, characterized by an increase in both fine and thick lateral root formation originating predominantly from nodal roots (**Figure 5b**). This change in root morphology rendered an expanded surface area and improved soil retention (**Figure 5b**), posing implications for enhanced nutrient and water absorption.

## Discussion

The increasing use of biostimulants in conventional agriculture accentuates the need for standardized regulation regarding product mode of action, especially when such mechanisms are not readily discernible (Du Jardin, 2015). A recommended method for elucidating biostimulant mode of action involves screening products in controlled environments using high-throughput phenotyping platforms (Rouphael et al., 2018). In line with this, the present study coupled automated high-throughput phenotyping, manual phenotyping, and estimation of photosynthetic potential (via a handheld phenotyping instrument) to determine the effects of FoliarBlend on rice growth in a controlled environment. FoliarBlend is a commercial prebiotic, a class of biostimulant with large potential to enhance crop productivity (Alahmad et al., 2023), The product contains >1, 000 small molecules (e.g., primary and specialized metabolites, lipids, electrolytes) derived from a proprietary consortium of beneficial microorganisms (https://agrigro.com/). FoliarBlend is formulated to provide growth/yield-enhancing benefits to both plant and soil, and was evidenced recently to modulate rhizosphere microbiome composition and enhance agronomic performance in soybean (*Glycine max*) under field conditions (Hale et al., 2024). The rice var. Kitaake was selected due to its suitability for growth in controlled settings and relatively short life cycle (~9 weeks; Jung et al., 2008), the latter of which enabled the efficient monitoring of FoliarBlend performance from seedling transplantation through plant maturation. The employed methodology collectively demonstrated that FoliarBlend increased seedling establishment, biomass accumulation, and photosynthetic efficiency under the defined conditions, and contributes to the broader understanding of biostimulant application and assessment in conventional agriculture.

The study first investigated treatment effects on seedling physiology using the LemnaTec Scanalyzer HTS system, a phenotyping platform that couples automated, non-invasive imaging with computer vision to reveal complex phenotypic traits (Chen et al., 2014). Such analysis is crucial, as seed germination and consequent seedling health are pertinent factors for rice yield (Yu et al., 2021) irrespective of crop establishment practice (Alam et al., 2020). Visible (RGB) imaging indicated that FoliarBlend consistently increased projected leaf area, convex hull area, caliper length, and compactness from 5 to 17 days after germination (DAG). These digital readouts are used commonly to infer stress adaptation and productivity in both mono- and dicotyledonous plant species (Ramírez-Rojas et al., 2022). Color classification of such images suggested consistent chlorosis (or absence thereof) among treatments for all but one-time interval.

Similarly, steady-state chlorophyll fluorescence, a measure estimated by exposing plants to a saturating light pulse and capturing the resultant electrical charge through a charge-coupled device and camera, exhibited no significant differences across treatments. This method is well-described by Baker (2008) and has been used to assess rice seedling responses to various abiotic stressors, such as salinity (Moradi and Ismail, 2007; Senguttuvel et al., 2014; Tsai et al., 2019; Faseela et al., 2020), light intensity (Faseela and Puthur, 2017; Faseela et al., 2020), flooding/submergence (Panda et al., 2006, Panda et al., 2008; Sone et al., 2012), polyethylene glycol, and heavy metals (Faseela et al., 2020). The uniformity of chlorophyll fluorescence readings between the control and FoliarBlend-treated plants implied that neither the management practices nor FoliarBlend application induced stress in the plants.

The FLUO and RGB imaging techniques were complemented by near-infrared (NIR) imaging, which leverages the strong reflection of NIR wavelengths by plant tissue. This is advantageous for evaluating leaf relative water content, a parameter significantly reflected in NIR spectra (Seelig et al., 2008; Fahlgren et al., 2015). The NIR values were similar among treatments, with those classified as “low” and “medium” exhibiting an increase over time. This could be attributed to the timing relative to the last watering event or to biomass accumulation, which in turn implies greater water utilization. The seven metrics concomitantly indicate that FoliarBlend facilitated seedling establishment relative to the control group without imposing additional stress.

When plants exceeded the capacity for automated phenotyping, they were transplanted into larger pots and subjected to manual phenotyping for the remainder of the study, with a second FoliarBlend application administered on the day of transplantation. The documented phenotypes included leaf number, plant height, and tiller and panicle abundance, with vegetative and reproductive parameters responding inversely to treatment (reduced vegetative growth; enhanced reproductive growth). In rice, tiller number is a fundamental determinant of final grain yield and enhances competition against weed species (Shahidullah et al., 2009; Pinson and Jia, 2016). The process of tillering involves the outgrowth of axillary buds from un-elongated internodes during the vegetative growth phase, leading to the formation of tillers, while buds formed on upper internodes after the vegetative phase typically remain dormant. Upon reproductive transition, the shoot apical meristem transforms into an inflorescence meristem, initiating panicle formation (Wang et al., 2015). This developmental shift underscores the relationship between tiller number and panicle production, with panicle number generally increasing alongside tiller number (Shahidullah et al., 2009).

However, this study presented a counterpoint to the typically positive correlation between tiller number and panicle number. Following FoliarBlend application, there was a decrease in tiller and leaf number, which paradoxically coincided with an increase in overall plant height, panicle number, aboveground biomass, and, most notably, grain yield. Aboveground biomass and grain yield were also increased in soybean upon FoliarBlend application (Hale et al., 2024). Ahmed et al. (2012) identified a negative correlation between rice plant height and tiller number, suggesting that an increase in height, as observed with FoliarBlend treatment, may naturally accompany a reduction in tiller number. Higher tiller density can increase competition among tillers, which in turn may contribute to tiller mortality, a phenomenon negatively correlated with the maximum number of tillers due to factors such as mutual shading and low straw nitrogen concentration, the latter of which may be exacerbated in controlled environments where nitrogen scavenging is limited (Wu et al., 1998; Schnier et al., 1990a, Schnier et al., 1990b; Matsushima, 1980; Tanaka et al., 1966).

Such dynamics suggest that prebiotic application might also mitigate competition for metabolic resources among tillers, thus promoting an increase in spikelet production per panicle. Heightened competition among tillers is associated with reduced spikelets per panicle, whereas a decrease in plant density, and hence competition, typically results in more spikelets per panicle under field conditions (Wu et al., 1998). Since spikelets per panicle are vital for grain yield, alongside the number of panicles per area (Wu et al., 1998; Counce and Wells, 1990), the present findings indicate that reducing tiller number could relieve metabolic competition among tillers, enhancing spikelet production per panicle and ultimately increasing grain yield. Future research must quantify tiller mortality in addition to abundance, quantify spikelet abundance, and should be conducted under field conditions to encompass pertinent environmental parameters (e.g., planting density, nutrient scavenging, and inter-plant competition).

Possible explanations to the relatively low seed set on the plants in the control group are due to both plant physiology and environmental control precision. In growth chambers, airflow and CO_2_ levels must be carefully managed. Poor air movement can limit pollen transfer, while CO_2_ enrichment can sometimes reduce pollen viability if not optimized (Yamori et al., 2014). When other variables such as light intensity, photoperiod, temperature, and humidity, are not optimized for rice, plants may produce fewer filled grains or show low seed set due to limited assimilate supply and impaired reproductive development (Köhl, 2015; Poorter et al., 2016; Hashida et al., 2022).

Measures of photosynthetic efficiency were taken from 49 DAG until the study’s conclusion using a MultispeQ 2.0. This handheld instrument encompasses a fluorometer, chlorophyll meter, and spectrometer for instantaneous, non-invasive determination of photosynthetic and environmental parameters (Kuhlgert et al., 2016). Furthermore, it has been used to characterize the impacts of biostimulants on numerous plant species, including durum wheat (*Triticum turgidum* L. subsp. *durum*) (Ben-Jabeur et al., 2021), grapevine (*Vitis vinifera* L.) (Ganugi et al., 2023), tomato (*Solanum lycopersicum*) (Carmody et al., 2020; Gitau et al., 2022), *Arabidopsis thaliana*, lettuce (*Lactuca sativa* L. cv. Finstar) (Senizza et al., 2023; Chovanček et al., 2023), cucumber (*Cucumis sativa*) (Shukla et al., 2023), common pea (*Pisum sativum*) (Shukla et al., 2024), and corn (*Zea mays*) (Martini et al., 2021; Nagarajan et al., 2024). In the present study, FoliarBlend application consistently increased Phi2, PhiNO, and relative chlorophyll content, with consistent reductions in PhiNPQ and NPQt. These patterns strongly reflect elevated photosynthetic efficiency in treated plants, which is likely associated with the observed increase in plant height (treatment plants in closer proximity to the light source in the growth chamber).

The elevated ΦNO observed with FoliarBlend treatment suggests an increase in non-utilized energy dissipation, potentially as heat or fluorescence, beyond the plant’s capacity for photochemistry (ΦII) or regulated heat dissipation (ΦNPQ) (Kramer et al., 2004). This scenario might arise if FoliarBlend boosts PSII efficiency to a point where the energy absorbed surpasses what the plant can immediately use or regulate, necessitating non-regulative dissipation (Kramer et al., 2004; Sedlacko et al., 2019). Moreover, the enhanced biomass and height of treated plants implies that FoliarBlend stimulated growth, possibly altering light interception and energy utilization dynamics. In a controlled chamber with constant environmental conditions, these effects might be amplified, evidencing how FoliarBlend influences not just photosynthetic capacity but also energy dissipation pathways, reflecting a balance between enhanced photosynthesis and the need to safely manage excess light energy. This indicates a putative role for FoliarBlend in modulating both photosynthetic efficiency and energy management, emphasizing the importance of understanding photosynthetic capacity *in situ* to grasp these dynamics fully.

## Conclusions

This study assessed the impact of FoliarBlend on the rice var. Kitaake from seed germination to seed production under controlled conditions. By integrating advanced high-throughput phenotyping with traditional manual phenotyping methodologies, it was evidenced that FoliarBlend application enhanced seedling vigor, biomass accumulation, photosynthetic efficiency, and grain yield. These findings highlight the potential of biostimulants to facilitate more sustainable and productive agricultural practices through improved crop performance at various developmental stages. Future work will expand upon this foundation by incorporating stress conditions into experimental designs and conducting field studies within actual production environments.

## Data Availability

The raw data supporting the conclusions of this article will be made available by the authors, without undue reservation.
